# Designing *for* and *with* People with Dementia using a Human Rights-Based Approach

**Published:** 2021

**Authors:** Shaan Chopra, Alisha Pradhan, Emma Dixon, Mary L. Radnofsky, Kausalya Ganesh, Amanda Lazar

**Affiliations:** College of Information Studies, University of Maryland, College Park, MD, U.S.; College of Information Studies, University of Maryland, College Park, MD, U.S.; College of Information Studies, University of Maryland, College Park, MD, U.S.; Advocate for the Human Rights of People with Dementia, Alexandria, VA, U.S.; College of Information Studies, University of Maryland, College Park, MD, U.S.; College of Information Studies, University of Maryland, College Park, MD, U.S.

**Keywords:** Human Rights, Dementia, User-Centered Design, CRPD, Heuristics

## Abstract

User-centered design is typically framed around meeting the preferences and needs of populations involved in the design process. However, when designing technology for people with disabilities, in particular dementia, there is also a moral imperative to ensure that human rights of this segment of the population are consciously integrated into the process and respectfully included in the product. We introduce a human rights-based user-centered design process which is informed by the United Nations Convention on the Rights of Persons with Disabilities (CRPD). We conducted two editions of a three-day-long design workshop during which undergraduate students and dementia advocates came together to design technology for people with dementia. This case study demonstrates our novel approach to user-centered design that centers human rights through different stages of the workshop and actively involves people with dementia in the design process.

## 1 INTRODUCTION

Once Human, Forever Human ^™^

I am, and shall always be a unique person with human rights equal to those of all others.

I’m also part of Humanity – the collection of all people who ever lived, are here now, and will ever exist.

At times, we assemble to create a whole greater than the sum of its parts, but we each maintain our individuality.

At times, I stand alone, different from everyone else, but still human.

Once human, forever human.

©2018 Mary L. Radnofsky

In User-Centered Design, technologies are typically created around the needs and preferences of participants. Ethics, which extend beyond individual preferences, are typically addressed in the context of seeking institutional review board (IRB) approval. Aside from the IRB process, the work of ensuring that research and resulting technologies do more good than harm is left to the varied ethical sensitivities of individual researchers [15].

We propose an alternative approach, which draws on human rights as a way to recognize moral imperatives in design. The United Nations enshrined these rights in the Convention on the Rights of Persons with Disabilities (CRPD) in 2006, affirming that people with physical, mental, or cognitive disabilities must be provided accommodation and adaptive environments by the state, so they may enjoy the same human rights as everyone else [7]. In this paper, we present a case study of an approach to user-centered design that draws on human rights throughout the process. We conducted two editions of a three-day-long design workshop in November 2018 and 2019. These workshops engaged undergraduate students in the user-centered design process of creating technology for and with people living with dementia. Two people with dementia, Mary Radnofsky^1^ and Diana Blackwelder^2^, joined researchers in teaching students how to weave principles of human rights into their technology designs.

## 2 BACKGROUND

At the end of WWII, the United Nations (UN) was created, and soon thereafter produced the Universal Declaration of Human Rights, to protect the inalienable rights of world citizens, based on principles of dignity, liberty, equal and fair access to justice, health, education, privacy, freedom of movement, self-expression, and the right to make one’s own choices. In 2006, the UN adopted the CRPD, mandating that people with physical, mental, or cognitive disabilities be provided accommodation and adaptive environments by the state, so that they may enjoy these same human rights. The CRPD is an international treaty based on international law and has been ratified by a majority of the member nations. In 2016, Mary Radnofsky became the first person with the disability of dementia to speak at the UN Conference of States Parties in New York and at the UN Human Rights Council Social Forum in Geneva later that year.

Dementia is a condition that involves changes in cognition and abilities, often affecting the ways that individuals engage in daily activities [35]. Dementia impacts memory, language skills, visual perception, problem solving, self-management, and the ability to focus, among other cognition, sensory and motor capacities [35]. Until the establishment of the CRPD in 2006 and the Equality Act [10] in 2010, dementia was not seen as a disability. Advocacy organizations such as Dementia Alliance International called for dementia to be recognized as a disability, stating that people with dementia should be “entitled to the appropriate disability support that any other persons or groups of disabled people are afforded” [16]. This view of dementia as a disability rather than a disease has a concrete impact on technology design [9, 24]. For example, the W3C Web Accessibility Initiative assembled a Cognitive and Learning Disabilities Accessibility Task Force [6] to develop cognitive accessibility requirements that include those with dementia. In this paper, we describe the role of two people with dementia as experts and advocates for the human rights of people with dementia, and the CRPD as a central resource in our user-centered design workshop.

## 3 PRIOR APPROACHES TO DESIGN WITH PEOPLE WITH DISABILITIES

“Universal Usability,” “Universal Design,” and “Design for All” have arisen to ensure that the needs of people with disabilities are met in all technologies, reducing obstacles to access, based on user abilities [18, 19, 25, 36]. Some argue that these approaches cannot sufficiently meet the diversity of human needs [13, 36]. A “design-for-one” approach has been suggested as an alternative [31] but can be difficult to generalize or scale [36]. To address these concerns, Wobbrock outlines a framework for ability-based design which focuses on what someone can do rather than on their disabilities, in the “universal application of ‘design-for-one”‘ [13, 36].

People with disabilities are often excluded from design activities and decisions about technology created for them. Building off the “design-for-one” approach, “Designing for User Empowerment” [21] includes users with disabilities throughout the entire design process, reflecting participatory action research methods [14], so “users of the technology are empowered to solve their own accessibility problems” [21]. Another approach, “Participatory Design,” includes future users as co-designers [34], who are treated as equals with researchers in co-creating [32] and have equal input in designing technological solutions [28]. In this way, participatory design addresses the human right to freedom of expression, democratizing the design process [22].

“Value Sensitive Design” seeks to account systematically for “universal concerns of ethical import” in the design process [11, 20], though it did not go so far as to reference human rights. Years later, researchers recognized inherent bias in a purported “universal set of values” [4, 23], leading Calvo et al. to call for evidence-based “design policy” and ethical frameworks, in addition to theoretical ones [5]. Consequently, Friedman expanded the concept to mean any values that individuals deem important in their lives [12]. Kirkham recently proposed incorporating parts of the European Convention on Human Rights (ECHR) into a “rights-sensitive approach” for technology design, because “decisions on values within design – especially controversial ones – are likely to have more legitimacy,” and because “some design decisions would have the force of law” [20]. In this paper, the CRPD served as our ethical and legal framework, while the user-centered design process inspired our framework for incorporating the human rights of people with dementia into technology design.

## 4 A HUMAN-RIGHTS-BASED APPROACH TO DESIGN

Below, we describe the four-step design process – setting the stage, gathering and analyzing data, generative design, and evaluation, highlighting where we introduced materials and content related to human rights. We use pseudonyms for students (see Table 1) and real names (with permission) for people with dementia and researchers.

### 4.1 Setting the Stage

Each workshop began with Mary introducing human rights from the perspective of people with dementia. She explained that most technologies are designed for caregivers, or in the absence of input from the person with dementia. For example, medical alert devices, which could be stigmatizing, are now often designed to look like jewelry, rather than a “medical device with a giant red cross.” In 2019, in addition to this overview, Mary prompted students to think about what they wanted in terms of their own basic human rights, generating responses such as “*equality*,” “*privacy*,” “*independence*,” and *“freedom of openly expressing [myself*].”

**Figure 1:**
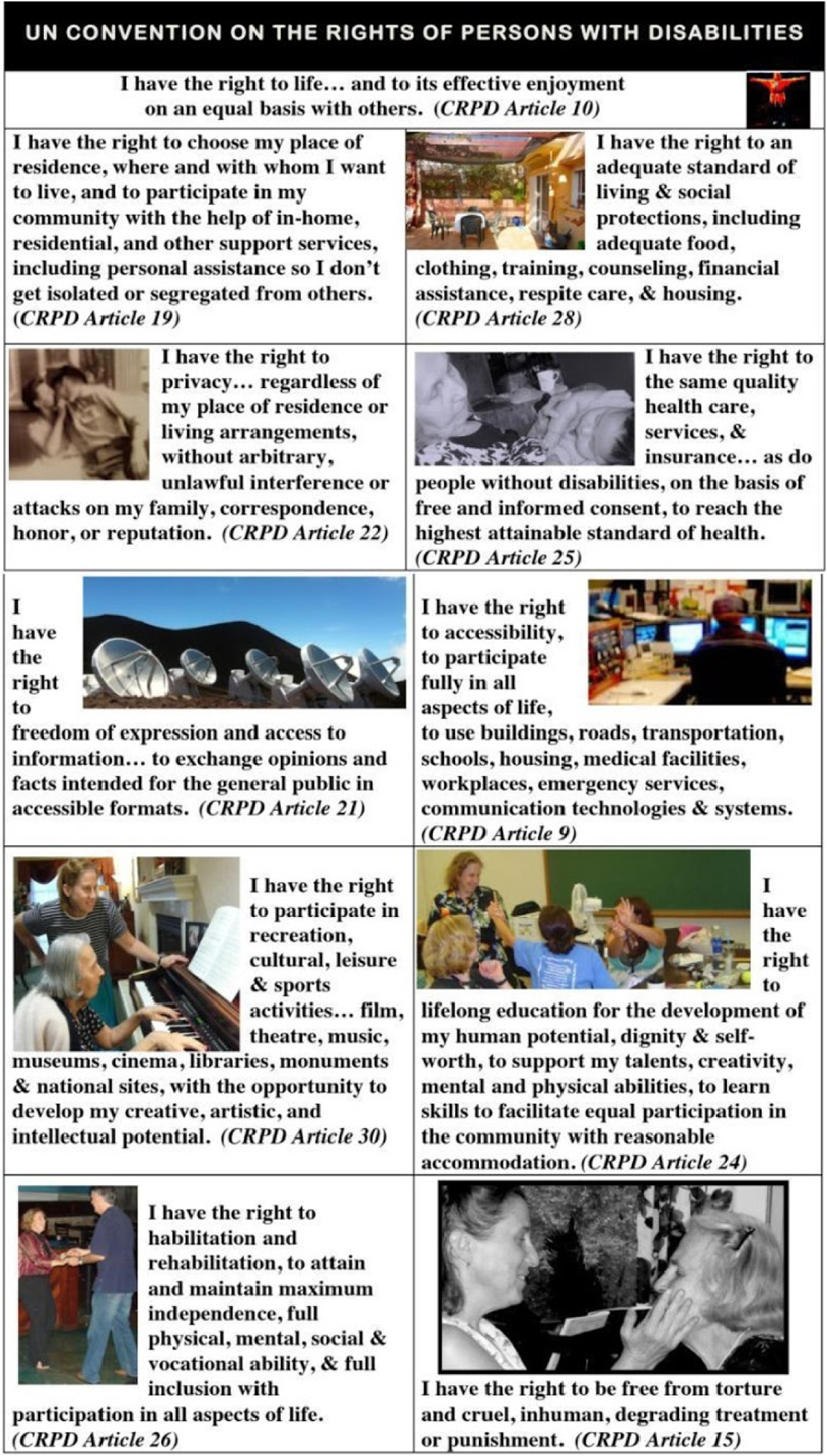
The Human Rights resource (©2018 Mary L. Radnofsky) based on the CRPD, distributed by Mary on day 1.

Mary also provided illustrated excerpts (see Figure 1) of the CRPD to highlight the treaty’s articles that applied to people with physical and cognitive disabilities such as dementia. Students referred to this resource throughout the design process.

Post data collection, students shifted to analysis. We taught them affinity diagramming, a process of organizing data into groups and themes [8]. While students took the lead on the process, Mary and Diana helped them reflect on insights from the affinity diagrams. In 2018, some themes that emerged were independence, safety, and control. In 2019, themes centered around fun, emotional intelligence, and self-determination.

### 4.2 Gathering and Analyzing Data

We taught students how to conduct qualitative interviews to directly learn from people with dementia about their needs. In 2018, the interviews were fairly brief. In 2019, students were mentored in more in-depth interviewing techniques, and given more time with the two dementia experts, to gather meaningful information. Diana’s group started with an idea related to falls, asking technology-focused questions: *“One of the ideas we were discussing regarding the smart mat…balance and falling can be an issue… and we can generate games, or some kind of exercise that is going to help [people with dementia] to keep their balance?”* (Maya). Emma redirected their questions, encouraging them to *“let the ideas come from the users.”* Diana also directed them by pointing out questions which were “too vague” (e.g., *“what do you struggle with”)*, and encouraged students instead to ask questions that are *“relatively general, but at least point me in a direction* so *that I can get my brain thinking about those things”* (e.g., Emma’s proposed question *“tell me about a particularly difficult time you had using a device”)*.

### 4.3 Generative Design

**Figure 2:**
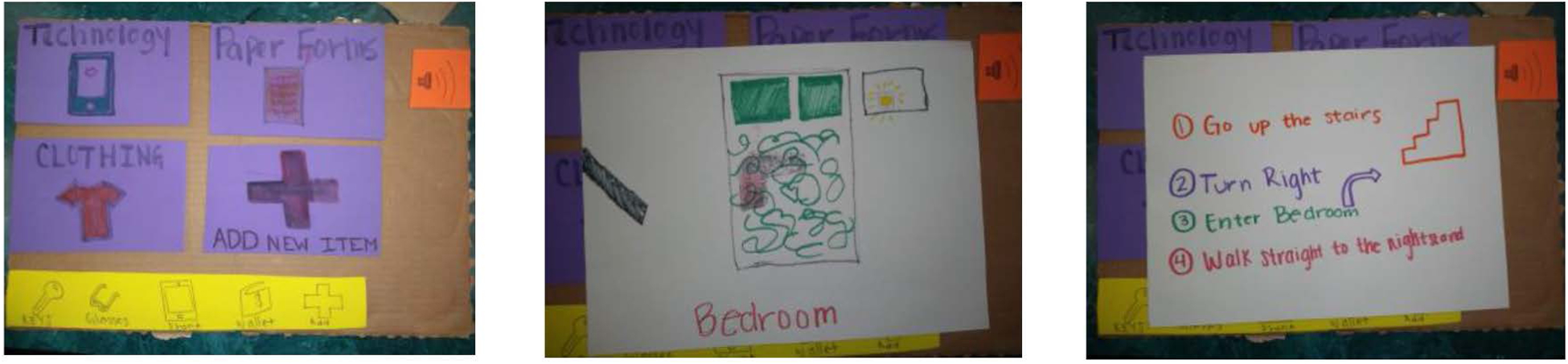
The goal of this 2018 prototype system was to assist people with dementia to locate things they had lost at home. Users select what they want to find, and the system provides the location of the item and directions to find it.

The next stage required creating and refining design concepts. Students sketched their initial ideas, then increased prototype fidelity using physical materials (e.g., cardboard, markers) and/or digital prototyping tools (e.g., In5Vision [17], Proto.io [30]). In 2018, students focused on the human rights of independence and autonomy that arose in their affinity diagram, to design technology that supports “home alone” experiences of people with dementia. Taylor noted that while some technologies can *“help you find your phone,” “there are a lot of other things you can lose track of,”* such as sunglasses. Mary agreed she could use a technology like this for tracking her keys or her service dog, Benjy, when he was off duty. Once this design opportunity was identified and students sketched *“futuristic, off-the-wall, and original ideas,”* they created a low-fidelity prototype (see Figure 2), wherein a user could put a tracking sticker on any item and log it into a system which could then direct the user to its location, when asked.

**Figure 3:**
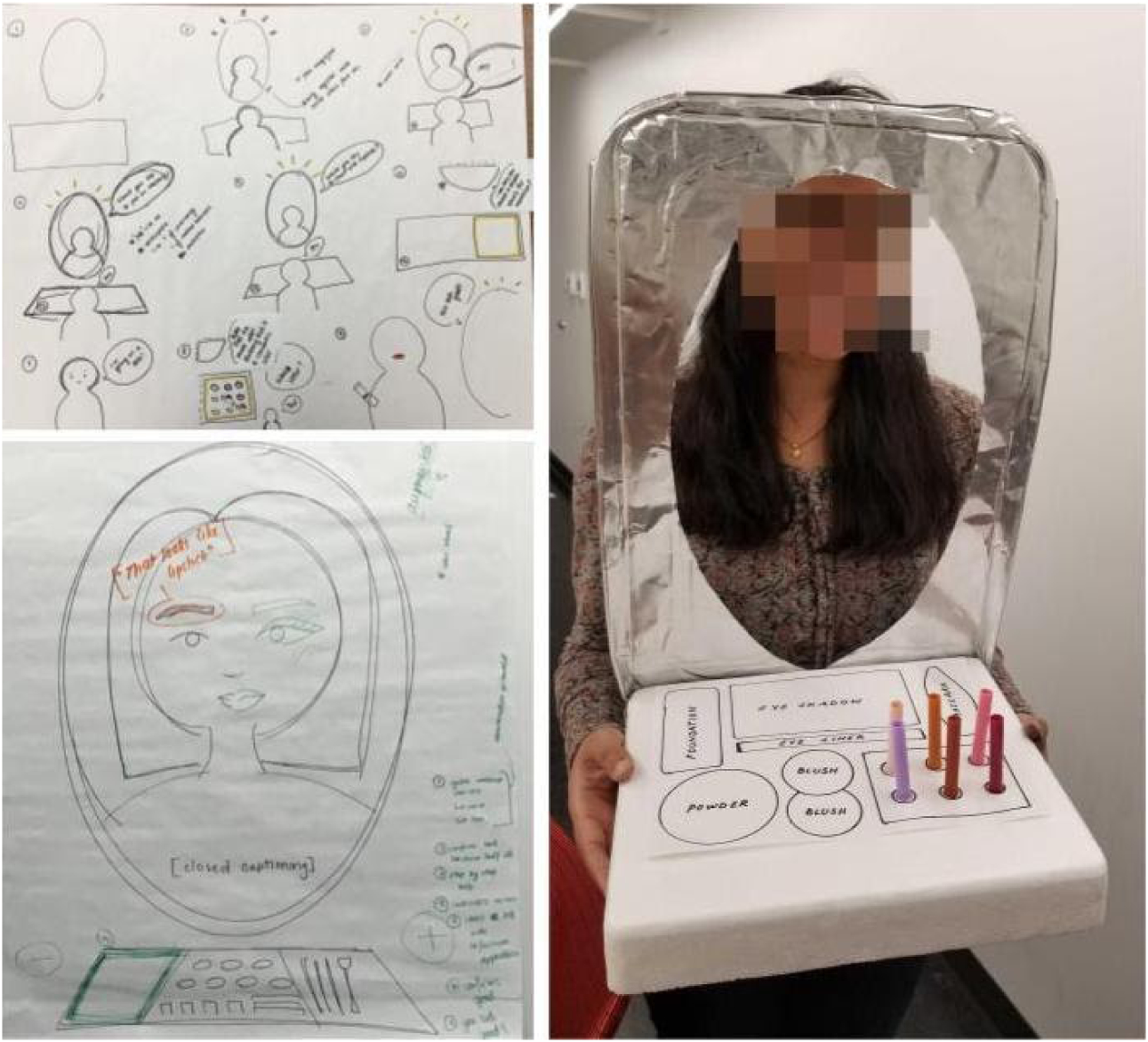
The “Smart Mirror,” created by Mary’s group in 2019 is a system designed to assist people with dementia in putting on make-up by helping them with decision-making through a series of interactive verbal and visual cues.

In 2019, ideas that emerged during the generative design phase included a reminder app (Briana), a context-sensitive conversational agent, like *“Alexa, that was very casual and kind of human”* (Maya), and a carpet that warns if *“you’re a little bit off-balance”* (Aruna). As students were ideating to create these prototypes, some of their sketches revealed how they considered social aspects of the technology. For example, Kira sketched the *“Smart Mirror”* that would help people apply make-up to correct areas of the face so they could feel confident going out in public. This idea was linked to the human rights theme of decision-making and self-determination that had emerged in affinity diagrams. The prototype for this idea (see Figure 3) uses an augmented reality feature to show how the person would look with different makeup styles. The base of the mirror – where makeup items are presented – was also designed to be assistive; depending on what the user chooses, that portion of the board would light up to indicate its position, with a verbal cue to pick it up.

**Figure 4:**
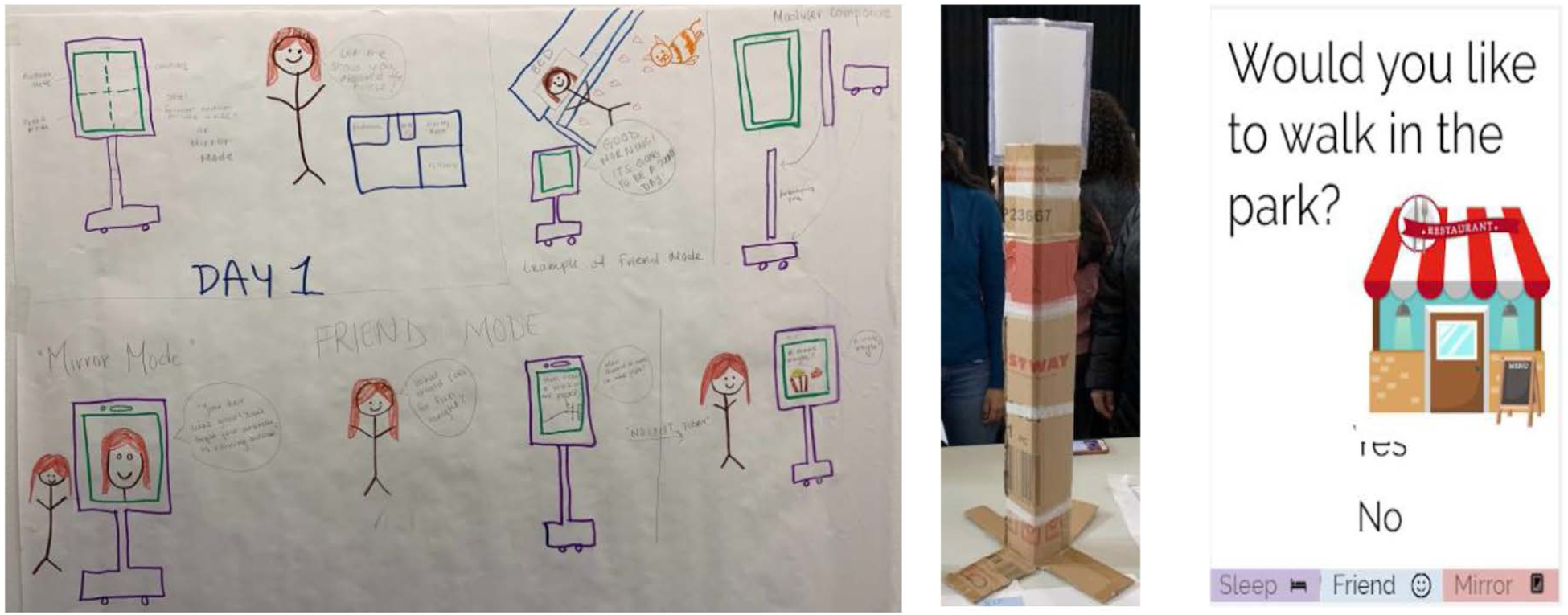
The 2019 “Buddy” prototype by Diana’s group was an assistive robot topped with an iPad. A digital prototype demonstrated different modes (sleep, friend, mirror) this robot could be put into.

The other design idea was an assistive robot (see Figure 4) called “Buddy,” which took into account users’ right to privacy. It would allow individuals to set the distance at which the robot should stay when following them, and to limit where the robot should not follow them inside their homes. They would also be able to select the robot’s working mode (e.g., sleep, friend, and mirror modes). For example, in “friend” mode, the user could question and interact with the robot in a casual manner, even using non-standard language.

### 4.4 Evaluation

We taught students two evaluation methods at the final stage of the design process. The first method was cognitive walkthroughs [29], in which people with dementia interacted with prototypes, asked probing questions, and provided constructive feedback. The second method was heuristic evaluation [27], which we refined over the two years of the workshop as described below.

In the first year, groups used the human rights resource to evaluate their designs in an *ad hoc* manner, thinking out loud to consider whether their designs took into account each of the human rights. In 2019, we co-created a printed set of human rights-based heuristics (See Table 2) with Mary, since heuristics are popular evaluation tools in HCI for comparing a designed system with a standard or “rule of thumb” [27].

The heuristic evaluation was done by independent project groups, each of which included a person with dementia, wherein they reflected together on aspects of their designs essential for preserving human rights. For example, Diana’s group discussed how CRPD Article 28 - *“Adequate standard of living & social protections”* – applied to their idea of an assistive robot. Initially, the students were unclear about the essence of this heuristic. They were trying to understand the significance of *“social protections”* for people with dementia, when Diana prompted them to also focus on the word *“adequate.”* She felt it important to understand different concepts of risk from which someone might require *“adequate”* protection. She explained further with an example of a caregiver who did not want the person with dementia *“to be going anywhere alone,”* an attitude that could impede an individual’s human rights, since everyone should be able to do things (e.g., going out alone) *“within reason”* and *“depending on their capability.”* The students decided that their robot should not impede this right but make suggestions through different privacy and autonomy configurations, since each individual with dementia may need different levels of autonomy over their decisions and actions.

The human rights heuristic sparked critical evaluation of the students’ technology designs once participants negotiated a shared understanding of the CRPD Articles. In both 2019 groups, each right was read aloud and interpreted, alternatively by the person with dementia and the students, until they all acknowledged agreement on what it meant. In HCI, heuristics check for violations, that is, whether an interface or system is, in any way, violating a rule of thumb [26]. One student, Aruna, explained that heuristics were *“the list of things that we have understood, that we should have incorporated into our designs… we should have taken these into account, into the creation of the invention, and the way [the researcher] is asking it, is more of an evaluative aspect.”* Diana also pointed out that a violation could raise the question as to whether the technology had created *“substandard living [conditions]”* for the person with dementia. Based on the heuristic’s different functions, Aruna, Kira, and Mary redesigned their heuristic worksheet by creating two columns, one for *“violated”* and one for *“included,”* adding space to explain how to provide a *“possible solution.”* Although both teams approached this phase differently, they demonstrated greater sensitivity to the rights of people with dementia after applying the human rights heuristics to their projects.

The students noted appropriately that not all heuristics were relevant to their projects. For example, Mary’s group felt that CRPD article 25 did not apply to their “Smart Mirror,” which did not involve healthcare. Thus, while the heuristic successfully encouraged groups to reflect on their ideas by designing with a human rights lens, there is a need to continue to investigate how they might best be integrated into and focus on various aspects of the design process, which we further discuss below.

## 5 DISCUSSION

This case study provides a first step towards answering past researchers’ call for practical [20] and evidence-based [5] ways to bring human rights into the design process [20]. Below, we reflect on our approach.

### 5.1 Incorporating Human Rights in Design Methodologies

Our approach seeks to systematically incorporate human rights into the design process. While existing design methodologies, such as Value-Sensitive Design [11], design for social acceptance [33], and an interdependence frame for design [2], touch on important aspects of human rights, they do not directly incorporate a human rights approach. We started the process by immediately introducing human rights and engaging students in discussions on how human rights apply to all lives. We provided them with human rights resource documents to which they could refer throughout the design process, and engaged them in activities (e.g., heuristic evaluation) so they could refine ideas as they applied their emerging understanding of equal access and accommodation for people with dementia. Students and people with dementia interacted throughout the course of the workshop so they could understand each other’s perspectives and design a technology that would best address the needs and wishes of people with cognitive disabilities.

The CRPD-based human rights heuristics were a key component of our approach to evaluating designs. Based on the traditional use of heuristics in HCI, we had originally created the human rights heuristics to check for violations [26]. However, when students and people with dementia used them, we discovered that these heuristics were more than a “violation checking” mechanism; they initiated discussions around human rights, encouraging students to critically reflect on their ideas and make conscious efforts to “include” one or more of the human rights at the heart of their designs. Based on these observations, we suggest that human rights heuristics should also question whether pertinent rights have been included and respected in a design, rather than merely check for violations. We also see opportunities to further investigate how human rights can be incorporated into other design practices such as card sorting (e.g., certain cards include human rights-based categories such as leisure and cultural activities).

While we see the potential for using these heuristics, we acknowledge critiques of how rule-based approaches to ethics may not address underlying structural and societal problems [1].

### 5.2 Facilitating Active Engagement with Human Rights

Throughout the workshops, students were taught to incorporate human rights principles as part of the design process rather than passively learn them as an abstract concept. People with dementia, students, and researchers worked together to explore human rights as a necessary part of the design process. Including people with dementia on the team not only gave students immediate access to end-users, but also demonstrated a commitment to the very human rights we were teaching – people with disabilities are equal (and valued) members of society, whose needs and rights must be directly addressed.

This involvement of people with dementia transcended their role as end-users in the user-centered design process. Mary and Diana played a key leadership role in the workshops, from helping create activities and materials (i.e., the heuristics) to setting the tone for the workshop (Mary’s introduction of the CRPD human rights). Mary and Diana were also more than co-designers; they served as teachers, leaders, mentors, and advocates for dementia rights.

Given how much the design process benefited from the technology, research, and advocacy experience of participants with dementia, we hope to investigate whether training in research practices might facilitate similar roles for other people with dementia. Yet, we recognize that people with dementia all have particular sets of skills and a lifetime of experiences and can serve many kinds of roles in user-centered design.

## 6 CONCLUSION AND DIRECTIONS FOR FUTURE RESEARCH

Eleanor Roosevelt, the first chair of the UN Commission on Human Rights, declared that human rights “carry no weight unless the People know them, unless the People understand them, unless the People demand that they be lived” [3]. In this case study, we have taken one step closer to consciously including and respecting human rights in technology design with and for people with dementia. Each person has individual needs but is also a human being with human rights equal to all others, regardless of disability. We have therefore created an environment in which people with dementia productively work and contribute in ways equal to their non-disabled peers, benefiting technology, themselves, and society. Our next steps involve further running remote design workshops, including people with dementia who cannot travel, with the goal of eventually having near equal numbers of people with dementia and students for optimum collaboration in the design process. Further, we are refining the activities involving human rights, and in particular the evaluation phase.

## Figures and Tables

**Table 1:** Participants included undergraduate students from US universities participating in an all-women and non-binary TECHNICA hackathon. In 2019, participants were divided into two groups. In 2018, all worked as a single group.

Year	Students at the Workshop				
2018	Casey	Taylor	Kristen	Erin	
2019	Maya	Briana	Muskan	Kira	Aruna

**Table 2:** The 2019 Human Rights Heuristics Evaluation form.

People with dementia have the human right to:	Is the standard violated? How?	Possible solution
Life, and enjoying it on an equal basis with others (CRPD Art. 10)		
Live and participate in a community of their choosing with access to support services (CRPD Art.19)		
Adequate standard of living and social protections (CRPD Art. 28)		
Privacy (CRPD Art. 22)		
Equal access to choose healthcare and related services (CRPD Art. 25)		
Opportunities for participating in cultural, recreational, leisure, and sports activities (CRPD Art. 30)		
Express their opinion and get information in formats most appropriate to them (CRPD Art. 21)		
Equal access to infrastructure, technologies, and systems (CRPD Art. 9)		
Education and lifelong learning for the development of their full human potential (CRPD Art. 24)		
Equal awareness of technologies that habilitate and rehabilitate to attain their maximum independence (CRPD Art. 26)		

## CCS CONCEPTS

• **Human-centered computing** → Human computer interaction (HCI); HCI design and evaluation methods; • **Social and professional topics** → User characteristics; People with disabilities.

## Footnotes

1 Mary is a human rights expert. She has been living with a degenerative brain disease for over 14 years and has a background in teaching education, human development, French, English, cyberethics, and research methods. She participated in both workshops and is a co-author on this paper.

2 Diana is an advocate for people with dementia. An electrical and information technology systems engineer, she was diagnosed in 2017 at the age of 55 with younger onset Alzheimer’s Disease, while still working in her profession. She was a part of the 2019 workshop.
